# Field trial of three different *Plasmodium vivax-*detecting rapid diagnostic tests with and without evaporative cool box storage in Afghanistan

**DOI:** 10.1186/1475-2875-10-169

**Published:** 2011-06-22

**Authors:** Amy FW Mikhail, Toby J Leslie, Mohammad I Mayan, Rohullah Zekria, Nader Mohammad, Mohammad A Hasanzai, Najibullah Safi, Christopher JM Whitty, Mark Rowland

**Affiliations:** 1 London School of Hygiene & Tropical Medicine, London, UK; 2 Health Protection & Research Organization, Kabul, Afghanistan; 3 HealthNet-TPO, Kabul, Afghanistan; 4 World Health Organization, Kabul, Afghanistan

## Abstract

**Background:**

Accurate parasitological diagnosis of malaria is essential for targeting treatment where more than one species coexist. In this study, three rapid diagnostic tests (RDTs) (AccessBio CareStart (CSPfPan), CareStart PfPv (CSPfPv) and Standard Diagnostics Bioline (SDBPfPv)) were evaluated for their ability to detect natural *Plasmodium vivax *infections in a basic clinic setting. The potential for locally made evaporative cooling boxes (ECB) to protect the tests from heat damage in high summer temperatures was also investigated.

**Methods:**

Venous blood was drawn from *P. vivax *positive patients in Jalalabad, Afghanistan and tested against a panel of six RDTs. The panel comprised two of each test type; one group was stored at room temperature and the other in an ECB. RDT results were evaluated against a consensus gold standard based on two double-read reference slides and PCR. The sensitivity, specificity and a measure of global performance for each test were determined and stratified by parasitaemia level and storage condition.

**Results:**

In total, 306 patients were recruited, of which 284 were positive for *P. vivax*, one for *Plasmodium malariae *and none for *Plasmodium falciparum*; 21 were negative. All three RDTs were specific for malaria. The sensitivity and global performance index for each test were as follows: CSPfPan [98.6%, 95.1%], CSPfPv [91.9%, 90.5%] and SDBPfPv [96.5%, 82.9%], respectively. CSPfPv was 16% less sensitive to a parasitaemia below 5,000/μL. Room temperature storage of SDBPfPv led to a high proportion of invalid results (17%), which reduced to 10% in the ECB. Throughout the testing period, the ECB maintained ~8°C reduction over ambient temperatures and never exceeded 30°C.

**Conclusions:**

Of the three RDTs, the CSPfPan test was the most consistent and reliable, rendering it appropriate for this *P. vivax *predominant region. The CSPfPv test proved unsuitable owing to its reduced sensitivity at a parasitaemia below 5,000/μL (affecting 43% of study samples). Although the SDBPfPv device was more sensitive than the CSPfPv test, its invalid rate was unacceptably high. ECB storage reduced the proportion of invalid results for the SDBPfPv test, but surprisingly had no impact on RDT sensitivity at low parasitaemia.

## Background

Over the last 15 years there has been a proliferation of malaria rapid diagnostic tests (RDT), which vary considerably in format, species detected and performance. As an aid to diagnosis in resource poor settings, malaria RDTs are increasingly being incorporated into national malaria management guidelines [1]. Diagnosis of malaria by clinical signs and symptoms alone is notoriously inaccurate; and where incidence is low or falling this results in high levels of over diagnosis and anti-malarial overtreatment in patients who do not have parasites and inadequate treatment in patients who do [2]. This has been extensively shown in sub-Saharan Africa including regions of holo and hyper-endemicity [3-7]. In South and Central Asia, which is predominantly a moderate to low transmission setting and where the majority of malaria is the product of two species (*Plasmodium falciparum *and *Plasmodium vivax)*, the proportion of patients incorrectly treated with an anti-malarial is likely to be high where laboratory diagnosis is available [8,9] and even higher where it is not [9]. The WHO Global Malaria Treatment Guidelines now recommend that all patients are treated on the basis of a laboratory-confirmed diagnosis [1].

RDT field trials undertaken in falciparum-predominant countries revealed five key operational issues with RDTs: (1) variation in quality between different manufacturers [10]; (2) variation in quality from the same brand but between lots [11]; (3) compromised performance of RDTs in field conditions due to heat and humidity exposure [12,13]; (4) geographic variation in RDT parasite capture rate due to local variations in malaria target antigens [14] and (5) reduced RDT sensitivity in patients with low parasitaemia [15,16]. The first two issues are related to manufacturing quality and are being addressed with the WHO/FIND malaria RDT evaluation programme [17,18]; RDTs entering the programme are required to have reached ISO manufacturing standards and are tested under standardized conditions by FIND with banked and cultured samples. The programme is currently falciparum oriented because non-falciparum parasites are difficult to culture *in vitro *and at a global level, falciparum malaria constitutes the greater threat to patient morbidity and mortality.

Identifying species is important; in most areas of co-endemic malaria, *P. falciparum *is resistant to chloroquine, which is still widely used to treat *P. vivax*. Mistreatment of *P. falciparum *with chloroquine virtually assures treatment failure, while treatment of *P. vivax *with more expensive artimesinin combination therapy (ACT) is effective [19], but wastes resources. Treating all undifferentiated fever as malaria (based on clinical signs) will lead to substantial wastage of drugs and misses potentially important non-malarial causes of fever, for example invasive bacterial infections [20]. Combination RDTs that can distinguish between *P. falciparum *and other species are increasingly favoured by national programmes in this region, principally because this distinction permits the restriction of expensive ACT to falciparum malaria alone, reduces the chance of treatment failure and improves treatment of non-malarial causes of fever. Other advantages include cost-effectiveness and improved ability to monitor disease trends. There is also increasing recognition of the capacity for *P. vivax *malaria to become clinically severe[21-23]. With over 70% of world's population in malaria endemic regions being at risk of *P. vivax*, this problem is far from trivial [24,25]. The ideal would be the universal deployment of effective point of care tests [1] that can rapidly identify all human malaria species and effectively target treatment for malaria and non-malarial causes of fever.

This study was conducted in Afghanistan, where malaria is predominantly caused by *P. vivax*, with *P. falciparum *a minority cause of disease. The country is hypo-endemic, with transmission varying widely by geographical area and season [26]. Most febrile illness is not caused by malaria, and most malaria is not caused by *P. falciparum *[27]. Within the Afghan healthcare system, malaria is currently diagnosed either by microscopy, or by clinical symptoms and signs alone. Currently 40% of Afghanistan's Basic Health Centres (BHCs) lack laboratories capable of performing malaria microscopy. This presents difficulties in meeting the requirements of revised WHO Malaria Treatment Guidelines. In the health facilities that currently lack a microscopy laboratory, RDTs may be the appropriate alternative.

Despite the importance of the disease, relatively few studies have specifically examined the ability of RDTs to detect vivax malaria. Studies that have been undertaken in the field indicate that the sensitivity of a number of vivax-detecting RDTs is greatly reduced at low parasitaemia [15,28,29]. Laboratory-based analyses also demonstrate that Plasmodium lactate dehydrogenase (pLDH) detecting antibodies are particularly vulnerable to heat degradation above 30°C [12,13]. Although only a proxy for variable-temperature field conditions, heat stability testing has thus far been typically conducted at FIND-certified reference laboratories, using incubators pre-set to four fixed temperatures. RDTs that have been stored in incubators between temperatures of 35 and 60°C for one month or more have shown either consistent performance throughout or reduced sensitivity to low parasitaemia after storage at the higher temperature (see [15,30] for example). It is, therefore, likely that pLDH sensitivity is causally linked to both temperature and parasite levels.

The performance and suitability of selected vivax-detecting RDTs under field conditions was investigated at the Malaria Reference Centre, Jalalabad, Afghanistan, with a focus on the practical challenges to vivax-predominant co-endemic regions: the diagnostic implications of heat-sensitive target antigens, the possibility of mitigating any heat damage with an appropriate technology that does not require electricity, and the impact of parasitaemia on the diagnostic accuracy of the tests.

## Methods

The principal objective was to evaluate the performance of three types of malaria RDT capable of detecting both *P. falciparum *and *P. vivax *in a controlled operational setting. The sensitivity, specificity and a measure of global performance for each RDT were examined across a range of representative levels of *P. vivax *parasitaemia found amongst outpatients. As a secondary aim, an evaporative cooling device was evaluated for its capacity to maintain or improve RDT sensitivity to *P. vivax *when compared to RDTs stored at ambient Afghan summer temperatures (38 - 45°C). Finally, each RDT was assessed to determine whether its performance under standard field conditions met WHO requirements for use in national programmes (sensitivity and specificity > 95%).

### Patient recruitment

Patients were recruited sequentially over a period of three months (June to August 2009) from seven outpatient facilities within a 5 km radius of the Malaria Reference Centre (MRC) in Jalalabad, where the evaluation of the rapid tests was conducted. The prevalence of vivax malaria amongst fever patients in the study area during the recruitment period was approximately 15%. Samples were kept in a cool box and transported to the MRC within two hours after collection from the patient. The panel of rapid tests were then conducted on the samples within 30 minutes of their arrival at the MRC.

Patients were recruited as "vivax positive" if they had a *P. vivax *positive clinic slide, as determined by the microscopists at each of the seven clinics during routine examination, while malaria negative patients were recruited on the basis of a negative clinic slide. In both cases, recruitment proceeded sequentially until the desired sample number for each group was reached. Approximately half of all the malaria cases selected with this protocol were children less than 10 years of age.

### Malaria rapid diagnostic test selection

The types of RDT used in the study were selected according to the following criteria of suitability for local and/or regional deployment: (1) Antigen detecting; (2) Ability to detect *P. falciparum *via the *P. falciparum*-specific HRP2 antigen; (3) Ability to detect *P. vivax; *(4) Ability to discriminate between *P. falciparum *and *P. vivax; *(5) Ability to detect other malaria species, if possible; (6) Easy interpretation of results for both species; (7) Strong performance under heat stress (exposure to 45°C or higher); (8) Strong performance in round 1 of the WHO-FIND malaria RDT product testing programme (if included).

Based on these selection criteria, three tests were chosen for the study: (A) CareStart™ 3-line Pf (HRP2) + Pan (pLDH) (Product no. G0131, AccessBio, New Jersey, USA); (B) CareStart™ 3-line Pf (HRP2) + Pv (pv-LDH) (Product no. G0161, AccessBio, New Jersey, USA); and (C) Bioline™ 3-line Pf (HRP2) + Pv (pv-LDH) (Product no. 05FK80, lot no. RDT9004, Standard Diagnostics Inc., Kyonggi-do, Korea).

The CareStart PfPan test was included because it scored highly in the first round of the WHO-FIND evaluations [17]. The study was conducted prior to the release of the round 2 evaluation results and the round 1 evaluation did not include any RDTs with vivax-specific test lines; however two models were included in this study because of the potential benefits of having a vivax-specific test line in a vivax-predominant area. The CareStart PfPv test was selected to determine if its performance would equal that of the PfPan RDT made by the same company. The Standard Diagnostics (Bioline) test was selected because the model is becoming increasingly easy to access throughout Asia, but the test had not previously been evaluated in an operational setting.

The integrity of the tests used in this study was ensured in the following manner: The temperature during transport was not monitored, but manufacturers were requested to ship the tests in insulated packaging with ice packs, in order to mitigate against exposure to high temperatures during transport. A single lot of each test type was provided specifically for this analysis and the tests were delivered two months prior to the start of the study, with an additional 11 months of validity remaining before they would expire in each case. Transit time for the RDTs from the manufacturer to the study site was five days for Standard Diagnostics and three weeks for the AccessBio tests. All tests were stored in identical conditions in a cooled warehouse in Nangarhar until one month prior to the beginning of the study, whereupon half the tests of each model were transferred to a room temperature cupboard in the study clinic and the remaining half were transferred to an evaporative cooling box.

### Cooling device selection criteria

Summer temperatures in Afghanistan can rise to over 45°C, exceeding the maximum storage temperatures for RDTs recommended by the manufacturers (30°C for AccessBio CareStart tests and 40°C for Standard Diagnostics Bioline). As most rural health centres do not have electricity, refrigerated storage or air conditioning is not an option. A locally appropriate cooling technology was therefore used. The target product profile for this technology was: (1) feasible to manufacture from local materials; (2) simple to install in clinics; (3) easy to maintain; (4) does not require electricity; (5) can maintain internal temperature below 30°C; and (6) remains effective up to an outside ambient temperature of 45°C and 75% relative humidity.

**Figure 1:**
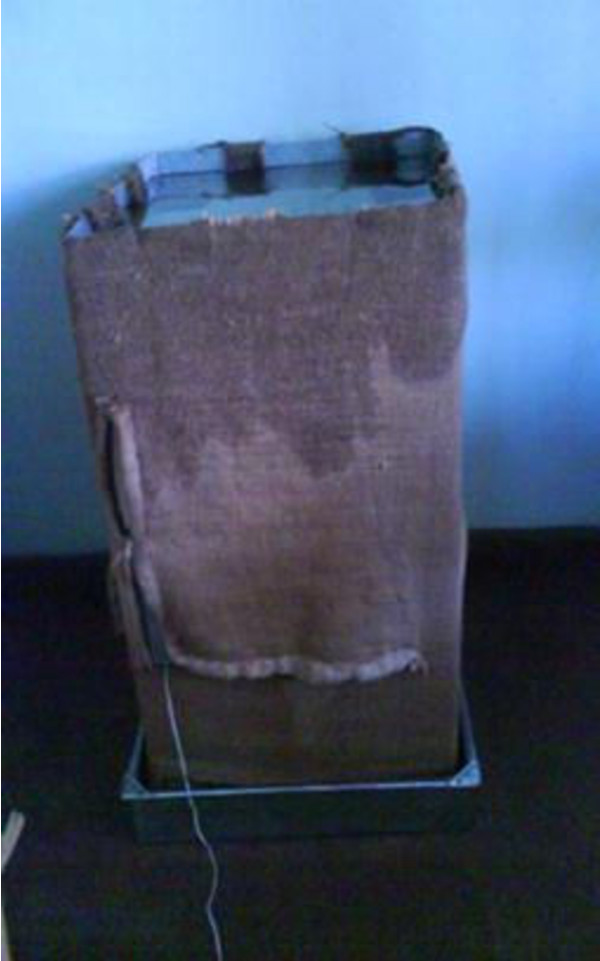
**Evaporative cooler box with drip tray**.

An evaporative cooling box adapted from designs tested by Bell and others in Cambodia [31] was chosen for this study (Figure 1). Pre-study enquiries indicated that health centres would prefer a portable box to underground pits, since the latter would require quite significant building work and be potentially disruptive to the layout of the clinic. Evaporative coolers are appropriate technology storage solutions which do not require electricity or much maintenance to run. The system consists of an enclosed metal box (the storage space) with extended sides to form a tray on the top capable of holding water. The box is covered in hessian, with material wicks connecting the hessian cover to the water filled tray. Water transfers by capillary action via the wicks to the hessian cover. Water evaporates off the hessian cover, cooling the metal box and its contents.

The box was manufactured to the specifications of the investigators by a local metal worker from aluminium, with a tailored hessian covering at a cost of USD $70 per unit. Unlike the Cambodian Cooler Box, wicks were tailored as a continuous extension to the hessian box covering, since this was found to improve the transfer of moisture from tray to box covering. Water in the upper tray was kept topped up to a predetermined mark. The performance of the prototype unit was evaluated prior to the start of the study by recording relative humidity and temperature inside and outside the box on an hourly basis for eight days. Thereafter, RDTs were stored in the evaporative cooler box and at room temperature for a minimum of one month prior to patient recruitment, resulting in a mean storage time in the study conditions of 2.5 months prior to use. The relative humidity and temperature within and outside the evaporative cooler box continued to be monitored throughout the study.

### Sample size

Sample size was calculated using the package epicalc, which is a component of the statistical open software, R [32]. The primary consideration for sample size was that it should be large enough to include patients with very low parasitaemia, since this has a known impact on RDT sensitivity. Parasitaemia less than 500 parasites per microliter can result in low sensitivity of pLDH based markers [28]. Recent trials in Afghanistan [33] show that 6-15% of vivax positive patients harbour < 500 asexual parasites/μL, and around half of patients have a parasitaemia < 5,000/μl (unpublished obs.).

Where studies have compared RDT accuracy at different levels of parasitaemia, the difference in sensitivity between different types of RDT at similarly low parasitaemia can be as little as 1% (SD Bioline vs. Optimal for *Plasmodium vivax *parasite densities less than 500/μL, [28]), 7% (SD Bioline vs. CareStart for *Plasmodium falciparum *[34]), or as much as 20% (Vistapan vs. Paracheck for *P. falciparum *parasite densities less than 100/μL [16]). The potential for each test to equal or exceed 95% sensitivity (WHO standard) at a typical low parasitaemia was the main outcome of this study; therefore the study was powered primarily to detect differences in sensitivity between low and high parasitaemia and between storage conditions, but not differences in sensitivity or specificity between tests.

A secondary consideration was the capacity of the RDTs to detect extremely low levels of parasitaemia in samples that were slide negative but PCR positive (i.e. where parasitaemia was below the detection limit of microscopy). Scant data exist on the proportion of false negative slides likely to be detected by PCR and it is difficult to generalize since this proportion depends on both the PCR method used, the quality of the blood films and the skill of the microscopist. An additional 20 clinic slide negative patients were recruited to examine this phenomenon.

To detect 6% hypo-parasitaemic patients (the proportion of the study population with parasitaemia less than 500/μL) with 95% confidence and an alpha of 0.05, a sample size of 241 *P. vivax *positive patients was deemed necessary. This figure was increased to 280 to allow for exclusion of any records that had missing data. In addition, a minimum of 20 malaria slide negative patients would be recruited, resulting in a total target sample size of 300.

### Patient recruitment (inclusion and exclusion criteria)

Patients were recruited over a three month period, from June to August 2009.

Patients were included if they were over five years, had given informed consent to participate (or where guardians had given consent in the case of minors) and were diagnosed by the clinic microscopist as *P. vivax *positive (target sample size = 280) or malaria slide negative (target sample size = 20).

Patients were excluded if they were anaemic (< 7 g/dL or equivalent clinical evidence), had any blood-based condition which would render blood sample collection risky, or were suffering from severe malaria as defined by WHO criteria.

### Sample collection and processing

Samples were collected from patients at participating clinics, as follows. After initial screening by the clinic microscopist, a further sample of blood was collected via finger prick to make a thick and thin reference slide. A venous blood sample of 3 mL was collected for the remainder of the tests, after which the patient's participation in the study was complete. Study diagnostic tests were not used in any treatment decision; patients were treated by the clinic doctor based on the results of the clinic slide and/or the clinician's judgment according to local guidelines. Venous blood samples were then transferred to the Malaria Reference Centre (within two hours of collection), where the remaining procedures were carried out. Approximately 10 μL of venous blood was spotted on to Whatman™ 3 MM chromatography paper (Whatman, USA), dried and stored for later processing by PCR. A second reference blood slide was made with venous blood and both slides were stained with 3% Giemsa and double-read blind to the other test results by two experienced microscopists in separate locations. Capillary and venous parasite counts were compared since the RDTs were tested on venous blood, but capillary blood is more typically used for both microscopy and rapid tests. Leukocyte counts were determined for each patient and used to calculate parasite counts per μL of whole blood in the thick film via standard methods [35]. A slide was declared negative if neither reference microscopist observed parasites after examination of at least 100 fields.

**Figure 2:**
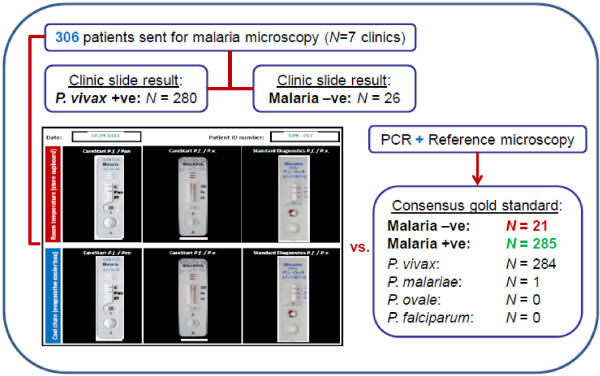
**Process flow chart of study design and patient recruitment**.

Finally, the six rapid tests (one stored at room temperature and one stored in an EC box for each model) were set out on a photographic panel (insert, Figure 2) and performed in each case according to the manufacturer's instructions. A plastic Pasteur pipette provided with the CareStart tests was used to transfer 5 μL of blood from the collection tube to the sample reception well on each test. For all CareStart tests, 2 drops of the relevant buffer were added to the buffer well; for the Bioline test, 4 drops of the provided buffer were added. Test results were read after 20 minutes. A quality control photograph was taken of each panel and later interpreted by one of the investigators (AFWM) who was blind to the original results. All laboratory results and patient data (age, sex and recruitment clinic) were recorded on pro-formas and double-entered blind into a Microsoft access database.

### DNA extraction from filter paper blood spots

Crude DNA was extracted from filter paper blood spots as previously described [36] but with the following minor modifications: spots were first washed in 500 μL PCR-grade water. The spots were then incubated at 95°C for 30 minutes in 150 μL of an extraction buffer comprising a 1:3 dilution of Tris-EDTA buffer, pH8. The crude DNA extract was stored in a -20°C freezer until use.

### Malaria confirmatory species identification PCR

PCR was performed on the crude DNA extracts as described by Mangold *et al *[37], with some adaptations to account for an alternative choice in kits and real time platform. Briefly, 5 μL of the crude DNA extract was added to 12.5 μL Absolute blue 2× SYBR green mastermix (ABgene, Surrey, UK), 5 μL nuclease free water and 2.5 μL of the consensus primer pair at a final concentration of 0.6 mM (Eurofins MWG Operon, Ebersberg, Germany). The reactions were transferred to an ABI 7300 real time PCR system with the following amplification programme: initial denaturation at 95°C for 15 minutes, followed by 40 cycles of 15 s at 94°C, 30 s at 50°C and extension for 30 s at 72°C. A dissociation curve was generated under the default ABI 7300 conditions. Species discrimination was achieved by determining the melting temperature peak of each sample relative to positive control peaks for *P. falciparum *(77.5 +/- 0.2°C)*, P. vivax *(81.5 +/- 0.6°C)*, Plasmodium malariae *(76.5 +/- 0.5°C)*, Plasmodium ovale curtisi *(79.6 +/- 0.2°C) and *Plasmodium ovale wallikeri *(79.1 +/- 0.2°C). Positive control DNA for each species was provided by C. Sutherland from the Malaria Reference Centre at the London School of Hygiene & Tropical Medicine.

### Data analysis

Data was double-entered into a Microsoft Access database and cross-checked with the Epinfo^® ^Data Compare module. Any discrepancies were corrected with reference to paper record forms. The dataset was analysed with the statistical software R^® ^(version 2.10.1) and Stata™ (version 8.2). A Wilcoxon signed-rank test was performed to determine if parasite levels in capillary and venous blood were different, while the level of agreement between pairs of reference results (two microscopy reads and PCR) was determined by Kappa values.

The sensitivity, specificity and global performance index of each RDT were determined using consensus reference results as the gold standard. The consensus standard was derived from both reference microscopy and PCR results without designating any single method as superior to the others, in a similar manner to that described by Boonma and colleagues [38]. A positive result from at least two of the three reference variables was scored as positive in the consensus variable and only samples that had negative results in all three reference variables were scored as negative. Samples for which only one reference result was positive were subjected to repeat microscopic examinations or PCR and the result generated was used as a tie-breaker.

As the RDTs investigated in this study were being considered for national deployment, a composite measure of global test performance was calculated, whereby in addition to separate indicators of performance such as sensitivity and specificity, the tests could also be compared with a single score. The global performance index (GPI) for each RDT was determined by expressing the number of true test results (both true positive and true negative) as a proportion of the total tested, thus incorporating sensitivity, specificity and the invalid rate in one unit. For the purposes of this calculation, invalid test results were not scored as true results but were included in the denominator (total tested). The three components were not differentially weighted in the formula as it was considered that each measure would be of equal importance to a national programme, albeit for different reasons.

Appropriate statistical tests of significance were used for the main outcomes; specifically, McNemar's statistic was used to compare the global performance index between tests stored in evaporative coolers and at room temperature and Fisher's exact test was used to determine the significance of parasite level effects on the global performance index of the RDTs.

### Ethics review

This study was approved by the Institutional Review Board of the Ministry of Public Health, Kabul, Afghanistan (IRB) and the ethics committee at the London School of Hygiene and Tropical Medicine. All participants or their guardians gave written informed consent. The study was designed and performed in compliance with Standards for Reporting of Diagnostic Accuracy Studies (STARD) guidelines [39].

## Results

In total, 306 patients were recruited. Of the 26 samples with a malaria negative slide at recruitment, 21 were confirmed as negative, but four were later determined to be infected with *P. vivax *and one with *P. malariae *according to the consensus reference standard (Figure 2). Of the 280 samples with a vivax positive slide at recruitment, all were confirmed to be infected with *P. vivax*. Seventeen records were excluded from parasite level analysis due to missing reference microscopy results. RDT results were scored as true positive, true negative, false positive, false negative or invalid according to the consensus gold standard (Table 1) and these groupings were used for all subsequent calculations.

**Table 1:** Numerical summary of results^$^

RDT type	Condition	True +ve	True -ve	False +ve	False -ve	Invalid
**CareStart Pf/Pan**	Room temp	271	19	0	4	12
	Cool chain	275	19	0	4	8

**CareStart Pf/Pv**	Room temp	258	20	0	23	5
	Cool chain	258	20	0	22	6

**Bioline Pf/Pv**	Room temp	223	19	0	10	54
	Cool chain	249	18	0	7	32

$ Designation by comparison with consensus gold standard, for 306 samples.

### Performance of the evaporative cooler box

**Figure 3:**
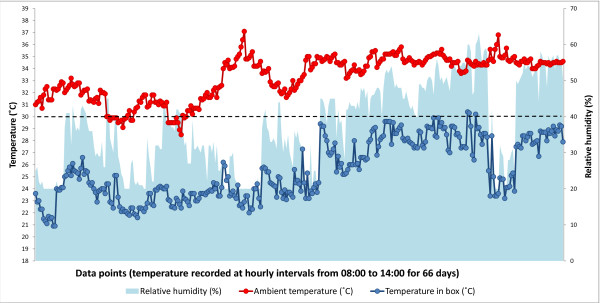
**Daytime temperature and humidity in the evaporative cooler box**. This box was installed in a room at the Malaria Reference Centre, Jalal Abad and the pre-trial data shown in this image was recorded from mid-May to early June 2009. Relative humidity outside the box is represented by the blue shaded area; room temperature is indicated by the red line and temperature inside the box is indicated by the blue line.

The evaporative cooler was examined for its capacity to protect tests, defined as the ability of the box to maintain an internal temperature of less than 30°C, when the ambient temperature is above 30°C. Initially, temperature differentials and relative humidity were assessed over an eight-day period prior to the start of the study. The box was placed in a typical un-cooled room inside the study clinic. Ambient temperature in the room ranged from 30 to 33°C, whereas temperature inside the EC box ranged from 20 to 26°C. Thereafter, temperature differentials in relation to relative humidity were continuously monitored during clinic hours (from eight in the morning until two in the afternoon) for the duration of the study (Figure 3). Temperature inside the box varied over a wider range than ambient temperature in the room and this was positively correlated with relative humidity (R^2 ^= 0.8, *P *= < 0.0001). Despite this variation, EC box temperature remained significantly lower than room temperature at all times; the average temperature differential was 8°C and ranged from 4 to 14°C. The highest ambient temperature (37°C) was recorded mid-study on 8^th ^July, at which time the corresponding temperature in the EC box was 23°C.

### Quality of the reference standard

RDT results were compared with a consensus gold standard, comprising the consensus result between two reference microscopy reads and the PCR result. The quality of each component in the gold standard was first verified by calculating the level of concordance and resultant kappa values for each pair of results. The two microscopy reads for both capillary and venous blood slides demonstrated high levels of agreement (98.98% and 98.96% respectively, with a kappa value of 0.93 in both cases). The level of concordance between each reference microscopy read and PCR results were lower (97%, with a kappa value of 0.85 and 6 non-concordant results in all four cases). The majority of non-concordant results (5/6) were from reference slides scored as negative that had a positive PCR result and presumably had sub-microscopic parasitaemia. The remaining sample (NHD_017) was positive for *P. vivax *on all four reference slides and all 6 rapid tests, but repeatedly *Plasmodium *negative by Mangold PCR. This sample was re-tested with three alternative PCR assays (Snounou nested PCR [40], Shokoples taqman PCR [41] and the Sharp taqman assay for detection of *P. vivax *[42]) by the malaria reference centre at the London School of Hygiene and Tropical Medicine; all three assays performed at the London School were also positive for *P. vivax*. PCR inhibition was identified as the likely reason for discordant results for this sample, since a crude extraction method was employed in Kabul that may not have been sufficient to remove all inhibitors, whereas a more robust Chelex resin extraction technique was used for the PCRs performed in London.

### Impact of blood source on parasite levels

To minimize discomfort for the patients, a single venous blood sample was collected from each donor and employed to evaluate the performance of the six rapid tests. Given that the more typical blood source for rapid tests is capillary, both capillary and venous blood slides were made for each patient and parasitaemia ranges from the two sources were compared with a Wilcoxon signed rank test. The analysis revealed a moderately significant difference between the two blood sources in one direction (*P *= 0.0059); parasitaemia levels were slightly higher in capillary blood than venous blood. Importantly, the venous blood used to evaluate the RDTs also had lower minimum parasitaemia levels than capillary blood (26 versus 175 asexual parasites/μL, respectively).

### Overall performance of RDTs

Both traditional comparative indicators for diagnostic tests (sensitivity and, specificity) and a composite measure of performance (the global performance index) were used in this study. With the caveat that only vivax positive and a small number of malaria negative samples were used, all three models of RDT demonstrated 100% specificity (95% CI 84-100). However, sensitivity and the global performance index differed between test models (table 2). The CareStart Pf/Pan test had the highest sensitivity (98.5%) followed by the Bioline Pf/Pv test (96.5%) while the CareStart Pf/Pv test was the least sensitive (91.9%).

**Table 2:** Global performance index and sensitivity of RDTs for P. vivax

RDT name	Storage	GPI^$ ^(% [95% CI])	Sensitivity (% [95% CI])
CS Pf/Pan	Overall	95.1 [92.7 - 97.5]	98.6 [97.2 - 99.9]
	
	Room temp	94.4 [91.9 - 97.0]	98.5 [97.2 - 99.9]
	
	EC box	95.8 [93.5 - 98.0]	98.6 [97.2 - 99.9]

CS Pf/Pv	Overall	90.5 [87.2 - 93.8]	91.9 [88.9 - 95.0]
	
	Room temp	90.5 [87.2 - 93.8]	91.7 [88.6 - 94.9]
	
	EC box	90.5 [87.2 - 93.8]	92.1 [89.1 - 95.1]

BL Pf/Pv	Overall	82.9 [78.7 - 87.1]	96.5 [94.3 - 98.7]
	
	Room temp	78.8 [74.2 - 83.4]	95.7 [93.2 - 98.2]
	
	EC box	86.9 [83.1 - 90.7]	97.2 [95.3 - 99.2]

$ GPI = (TN+TP/*N*) × 100 where TN, true negative; TP, true positive; *N*, total tested.

**Figure 4:**
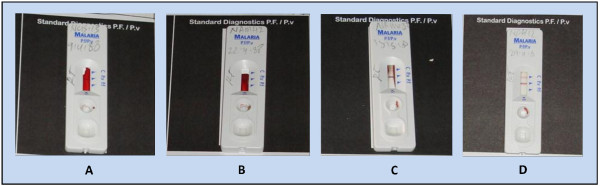
**Examples of invalid RDT results**. Photos of invalid RDTs (A - C) and one functional RDT (D). Panel A shows error type 1 (blood did not clear after 20 minutes); Panel B shows error type 2 (control line not reached by the sample after 20 minutes); Panel C shows error type 3 (control line did not appear after 20 minutes).

When the rapid tests were compared by their global performance index, the CareStart Pf/Pan RDT maintained its lead position (GPI 95%) followed by the CareStart Pf/Pv test (GPI 91%) and the Bioline Pf/Pv test (GPI 83%). The lower GPI of the Bioline RDT was attributable to the high number of invalid test results in this model (Figure 4).

### Impact of EC box storage on the global performance index

There were no significant differences in sensitivity between storage conditions for any of the three RDT models (Table 2). However, the global performance index for the Bioline test was significantly higher (*P *< 0.001) in the group stored in the EC box than for those stored at room temperature. This difference was attributable to the invalid result rate; while Bioline tests held under both storage conditions yielded invalid results, EC box storage resulted in a reduced number (31 in the EC box versus 52 at room temperature). Most of the invalid test results were due to the poor flow of blood along the device, which often failed to reach the control line within the test period (Table 3, Figure 4).

**Table 3:** Causes of rapid test invalidation^$^

Test type	Storage type	Type 1^a^ N (%)	Type 2^b^ N (%)	Type 3^c^ N (%)
CS Pf/Pan	Room temp	0	2 (100)	0
	EC box	0	0	0

CS Pf/Pv	Room temp	0	1 (100)	0
	EC box	1 (50)	1 (50)	0

BL Pf/Pv	Room temp	17 (33)	23 (44)	12 (23)
	EC box	13 (42)	13 (42)	5 (16)

$ Not including tests invalidated by operator error

a Type 1: Blood not cleared

b Type 2: Control line not reached

c Type 3: Control line absent

### Impact of parasitaemia level on RDT performance

Previous studies on *P. vivax *detecting rapid tests have shown that test lines are increasingly likely to be false negative if parasitaemia is less than 5,000/μL or less than 500/μL, while the WHO/FIND evaluations define low parasitaemia as less than 200 asexual parasites per microliter. To ascertain if low parasitaemia affected RDT performance in the present study, all three cut-off points (<>200/μL, <>500/μL and <>5,000/μL) were initially considered. Since the proportion of samples with parasitaemia less than 500/μL was very small (N = 4), samples were divided into low (<5,000/μL) and high (>5,000/μL) parasitaemia groups for subsequent analysis. All performance parameters (sensitivity, invalid result rates and the global performance index) were compared for both groups.

Performance in two of the three RDTs (CareStart Pf/Pv and Bioline Pf/Pv) were adversely affected by low parasitaemia. For the CareStart Pf/Pv test, the global performance index and sensitivity were significantly reduced when parasitaemia levels fell below 5,000/μL. There was a 16% difference in GPI (*P *= < 0.0001, table 4), while sensitivity decreased from 97.5% to 82% in the low parasitaemia group.

**Table 4:** Effect of storage condition and parasite level on RDT global performance index

Rapid test name	Storage	< 5,000 T/μL	> 5,000 T/μL
CareStart Pf/Pan	Room temp	94.1 [89.8 - 98.4]	96.8 [94.1 - 99.6]
	
	EC box	95.8 [92.1 - 99.5]	98.10 [96.0 - 100.0]

CareStart Pf/Pv	Room temp*	81.5 [74.4 - 88.6]	98.1 [95.6 - 100.0]
	
	EC box*	82.4 [75.4 - 89.3]	97.5 [95.0 - 99.9]

Bioline Pf/Pv	Room temp	82.4 [75.4 - 89.3]	76.6 [69.9 - 83.3]
	
	EC box	90.8 [85.5 - 96.1]	85.4 [79.9 - 91.0]

* Significant difference between false result rates for low and high parasitaemias.

For the Bioline Pf/Pv test, the proportion of invalid results increased by 10% in the EC box and 12% at room temperature in the high parasitaemia group (> 5,000/μL) when compared to the low parasitaemia group (*P *< 0.01). The resultant differences in GPI were smaller and non-significant because there were also a number of false negative RDT results in the low parasitaemia group (5 in the EC box and 7 at room temperature, respectively) but none in the high parasitaemia group (Table 5).

**Table 5:** Effect of parasite level on rapid test results

RDT type		Rapid test results			Slide results
		
	Storage	Invalid	Negative	Positive	Parasitaemia
**CS PfPan**	Room temp	1	22	1	Negative
		6	1	112	Low ^a^
		5	0	153	High ^b^
	
	EC box	1	22	1	Negative
		4	1	114	Low
		3	0	155	High

**CS PfPv**	Room temp	0	23	1	Negative
		3	19	97	Low
		2	1	155	High
	
	EC box	0	23	1	Negative
		3	18	98	Low
		3	1	154	High

**BL PfPv**	Room temp	2	22	0	Negative
		14	7	98	Low
		37	0	121	High
	
	EC box	3	20	1	Negative
		6	5	108	Low
		23	0	135	High

^a ^Low parasitaemia: < 5,000/μL;

^b ^High parasitaemia: > 5,000/μL

## Discussion

In this study, the performance of three multiple species combination rapid tests for malaria were evaluated in Afghanistan against a typical sample of patients presenting with a representative range of different levels of vivax parasitaemia; the results revealed clinically important differences between RDT models. An evaporative cooler box was simultaneously evaluated to determine if this appropriate technology storage device would prevent heat-induced deterioration in test sensitivity or global performance index under operational conditions. The unit proved to have a positive impact on some test models, while surprisingly it did not improve sensitivity to low parasitaemia.

### Evaporative cooler box performance

As *P. vivax*-detecting RDTs are known to be particularly vulnerable to heat damage, their effective deployment for national programmes is dependent on the provision of locally appropriate cool chain storage solutions. In this investigation, evaporative cooling technology was selected for evaluation as it does not require electricity, could be made locally, requires little maintenance and could be placed anywhere in the clinic. This cooling system is also familiar to local end users, since the same technique is used to keep water pitchers cool during the summer.

A preliminary objective of this evaluation was to determine whether a locally made evaporative cooling box could maintain an internal temperature within the range recommended for storage of RDTs by manufacturers (typically between 15 and 30°C). This was realized, since throughout the study, temperature within the box never exceeded 30°C, even though the maximum temperature recorded outside the box during the study period was 37°C. As expected, increasing daily temperatures did not adversely affect the ability of the cooler to maintain significantly lower internal temperatures, but increasing relative humidity did reduce the cooling capacity of the box, resulting in smaller temperature differentials. These results correspond well with the findings of a Cambodian pilot study on evaporative cooler boxes of a similar design [31]. Because on average relative humidity in Afghanistan is low and does not exceed 70%, a higher average temperature differential can be achieved that would work in favour of this technology in the Afghan setting. During the study, the maximum temperature differential achieved was 14°C (RH 20%).

Additionally, the EC box was tested in eastern Afghanistan, where summer temperatures typically exceed 40°C and are representative of the hotter, low-lying regions in the country where most malaria transmission occurs. Winter temperatures were not considered in this investigation, although they could potentially cause difficulties as during this season most provinces experience sub-zero temperatures, particularly at night. Alternative mechanisms would have to be considered to protect RDTs from freezing during the winter months.

The principle objective was to ascertain if the evaporative cooler box could mitigate or prevent heat-induced deterioration in rapid test performance. To examine this, all performance parameters were compared for tests that had been stored at room temperature versus those that had been stored in the EC box. However, the impact of the box on test performance cannot be understood without prior reference to the individual characteristics of the rapid tests; a contextual overview of the performance characteristics of each RDT is, therefore given below.

### CareStart PfPan

The CareStart PfPan test detects *P. falciparum *HRP2 with falciparum-specific antibodies in the first test band and *Plasmodium *species lactate dehydrogenase (LDH) with pan-specific antibodies in the second test band. Of the three RDTs evaluated, this test was the only device that met and exceeded WHO standard requirements for national deployment, irrespective of pre-use storage conditions and sample parasitaemia levels (sensitivity, specificity and global performance index were above 95%). The control line failed to appear in only two out of 612 tests, both of which had been stored at room temperature.

The test produced one false negative result (under both storage conditions), for a sample which was later determined to be infected with *P. malariae *and had the lowest parasitaemia of all the samples tested. The *P. malariae *infection in this sample was initially detected by Mangold PCR, as one reference read reported both blood slides to be negative and the second misidentified the infecting species as *P. vivax*. Species identification was later verified by the corresponding author, who located two characteristic band form trophozoites in the thin film. *Plasmodium malariae *is extremely rare in the region, but has been documented previously [43] and may be under-reported due to the difficulties of identifying this parasite with typical clinic microscopy, where the thin film is rarely viewed [44].

As such, the very low parasitaemia level of this sample was the most probable cause of detection failure; the parasites were also missed by one of the reference microscopists and the real-time PCR signal crossed the threshold at 37 cycles, indicative of a very low concentration of target DNA. Of the published evaluations on pan-specific LDH detecting RDTs, very few have reported on their ability to detect *P. malariae*; where this has been examined, sensitivities for the parasite range from 30 [45] to 45% [46] and in the latter case, poor sensitivity was attributed to the low parasitaemia levels typical of this species.

At the time of writing, there are seven published studies that have reported on the use of the CareStart Pf(HRP2) Pan(pLDH) test to detect *P. vivax*. Of these, two were field studies conducted in Uganda [16] and Madagascar [34], where the number of vivax cases was insufficient to draw any conclusions about this species; a third study consisted of a large school-based survey in Ethiopia, where test sensitivity was not reported [47]. An additional two studies conducted in reference settings reported sensitivities of 77.6% [45] and 79.2% [48]; in both cases considerably lower than that determined in the present study. Field studies conducted in Myanmar [15] and Ethiopia [30] also report lower sensitivities of this RDT to *P. vivax *(78.5% and 85%, respectively), although in both cases the authors note that the CareStart test was more sensitive to *P. vivax *than the other RDTs they had tested in parallel.

There are a number of possible reasons for the higher sensitivity determined in this study. One key distinction between field and reference settings is that in the field, the source of blood is fresh, whereas studies conducted in reference settings typically use frozen samples, where repeated freeze-thaw cycles may have damaged some of the antigens resulting in artificially reduced test sensitivity. A second potential factor is antigen variability; although LDH antigens are generally thought to be less heterogeneous than HRP2, a small amount of variation has been detected (see [14] for example). The samples used in the present study were collected from a single region in eastern Afghanistan and are therefore likely to have low within-sample variation.

Lastly, these differences may be attributable to the different proportions of hypo-parasitaemic patients in each study. For example, 28/254 samples with parasitaemia < 500/μL were evaluated with the pan-specific model in Ethiopia, as compared to only 4/280 samples with this level of parasitaemia in the present study. Patients with very low parasitaemias may have been under-represented, as the main criterion for enrolment was a vivax positive slide as determined by clinic microscopists with varied levels of expertise. This may have reduced the scope for recruiting a sufficient number of hypo-parasitaemic patients; none the less, a sensitivity of 98.6% may be considered representative for this RDT where sample parasitaemia is greater than 500 parasites per microliter.

### CareStart PfPv

The CareStart PfPv test detects *P. falciparum *with falciparum-specific HRP2 antibodies and *P. vivax *with vivax-specific antibodies to lactate dehydrogenase. Although this test also demonstrated 100% specificity, its global performance index was lower than the other two RDTS, due to its reduced performance at parasite densities below 5000/μL. Reduced sensitivity at this level of parasitaemia is not unusual in *P. vivax *detecting RDTs; field evaluations of the Standard Diagnostics FK70 RDT and ICT malaria Pf/Pv yielded reductions in sensitivity of 19% and 26% below 5,000/μL, respectively [28,29]. In the present study, test sensitivity was also markedly reduced from 98% to 82%. Sixteen samples were scored as false negative under both storage conditions, with parasite densities ranging from those undetectable by microscopy (with a positive PCR result) to 3261/μL. A further seven and five RDTs, respectively, were only false negative under one storage condition (room temperature or evaporative cooler). There was no discernable pattern to the results falling within this group, which can be interpreted to mean that parasite densities below 5,000/μL reduce the reliability and accuracy of this test, but do not always result in false negatives.

At the time of writing, the CareStart Pf/Pv RDT has been assessed in the second round of the WHO/FIND evaluation [18] and in two published field studies, which were carried out in Ethiopia [49], [50]. Although the WHO/FIND evaluation was carried out in a non-operational setting, a drop in global performance index of 10% from standard (> 2000/μL) to low (< 200/μL) parasite densities was observed, indicating that the test performed better at low parasitaemia than in this investigation. This may have been due to the more controlled setting in the latter study, or the limited number of source sample materials utilized.

Both Ethiopian field studies found the overall sensitivity to *P. vivax *to be higher (95.3% in Jimma and 99% in Wondo Genet) than was found in the present investigation; however a complete breakdown of parasite densities in the study population was not reported in either case. A small number of false negatives were identified in Jimma, where the authors cite parasitaemia < 100/μL as the reason for non-detection. Because this RDT detects *P. vivax *specifically, it is unlikely to be used in regions where the number of vivax cases is negligible. Further evaluations of this test in other regions are needed to determine if the problems identified here remain an issue outside the current study setting. However, recent results from a multi-site randomized trial of this device in clinics situated in the same province show consistently reduced sensitivity at low parasite densities, with end users reporting a perception that the test is sometimes unreliable as compared to other methods (Leslie et al., unpublished results).

Interestingly, storage of this test in the evaporative cooler had no impact on sensitivity to low parasite densities. A link between poor sensitivity to a low parasitaemia and heat damage has previously been documented for some RDTs [12], although there were no *P. vivax *samples in the test panel, nor in the heat stability tests conducted as part of either of the recently completed WHO/FIND evaluations [17,18]. A literature search yielded only a single reference to reduced sensitivity for *P. vivax *low parasitaemia after heat exposure, in the ICT Pf/Pv device [30]. The lack of information on this interaction is partly due to the difficulty of culturing *P. vivax *parasites for stability testing; it is also difficult to obtain standardized parasite densities from wild samples. In general, though, the relationship between heat, parasitaemia and RDT performance appears to be more complex than previously thought - even for pan-specific detection of *P. falciparum *LDH - and may only have relevance for a limited selection of RDTs. The cause of the reduced sensitivity to low parasitaemia for this test remains unclear and merits further investigation.

### Bioline PfPv

The Bioline PfPv test detects *P. falciparum *HRP2 with the first test line and *P. vivax*-specific LDH with the second test line. As with the other two devices in this evaluation, test specificity was 100%. Of the two vivax-specific devices, the Bioline test was more sensitive (maintaining sensitivity above 90% under both storage conditions and irrespective of parasite density). However, the test produced an unusually high number of invalid results, which resulted in a global performance index of only 82.9%.

In addition to inclusion in round 2 of the WHO/FIND evaluations, the Bioline PfPv test has been subjected to a single trial in a reference setting [51]. To date, no other studies have been conducted on this test in the field. Sensitivity to vivax parasites in the reference setting was lower than that reported here (75.8%), with a significant reduction in sensitivity to parasite densities < 500/μL. A single test line, vivax-specific LDH device made by the same company has also been evaluated in a reference setting [52] and in Korea [28], with overall sensitivities of 88% and 93.4%, respectively. Reduced sensitivity to low parasitaemia is reported in both cases; however over 40% of patients recruited in Korea had parasite densities below 500/μL, making it difficult to compare with the higher parasite levels seen in the Afghan setting.

Although an unusually high number of invalid tests were recorded in the present study, the phenomenon has received scant attention elsewhere. Invalid result rates for rapid tests may be under-reported, because the traditional parameters used to measure the performance of diagnostic tests (sensitivity, specificity, negative and positive predictive values) do not take invalid results into account. Moreover, during field trials, operators are typically instructed to disregard invalid results and repeat the tests; unless specific mechanisms are instigated to record them, the data is lost. In the current investigation, photographs of the rapid tests were taken 20 minutes after sample and buffer deposition, allowing for easy discrimination between operator errors and test failure, as well as facilitating characterization of the reasons for an invalid result. In some cases, the blood did not clear from the background (rendering the test result illegible), while in others the blood sample had stopped progressing up the strip and failed to reach the control line. Notably, this is similar to observations for the Bioline single band vivax-specific LDH test in the reference setting [52]; the authors of that study also described difficulties with the progress of both buffer solution and sample up the strip on two occasions.

The proportion of invalid results was affected by both parasitaemia levels and storage temperatures. Contrary to expectations, the test performed worse at parasite densities greater than 5,000/μL, exhibiting almost twice the number of invalid results. The Global performance index was lowest for tests that had been challenged with highly parasitaemic samples and stored at room temperature.

The maximum parasite density for an invalid result was 28,181/μL and PCR cycle thresholds consistently fell below 30, indicative of the high levels of parasite DNA in these samples. A plausible explanation for this may be that the viscosity of the blood samples increased with increasing parasitaemia [36], thus restricting sample flow up the device and preventing the sample from reaching the control area, causing an invalid result. Although host-cell clumping is a phenomenon typically associated with *P. falciparum*, one study has also documented both increased cell rigidity and clumping behaviour in erythrocytes infected with vivax parasites [53]. Both these factors would increase the viscosity of infected blood, in proportion to parasitaemia levels.

The Bioline PfPv test was the only device assessed in this study for which storage in the evaporative cooler box had a notably positive effect on performance. The invalid rate was significantly reduced when the tests were stored in the EC box. It is possible that warmer room temperatures may have resulted in some kind of mechanical damage, such as warping, to the device. If this were the case, it could explain why the flow of the sample up the strip was either curtailed completely, or slower than the time allotted for clearance of the test window by the manufacturer (maximum 30 minutes).

### Applicability of the study design for national programmes

The findings of the present study were used by the National Malaria and Leishmaniasis Control Programme to identify a suitable RDT for country-wide implementation. Three features of the study design proved to be particularly suitable in assisting with the decision making process.

Firstly, parasite positive patients were deliberately chosen as the focus of this study, which resulted in a wide and representative range of natural parasite densities. Slide positivity rate in Nangarhar rarely exceeds 20%; thus a much larger sample size would have been necessary to recruit the equivalent number of positive cases if all fever patients who presented at the clinics had been enrolled. One potential draw-back of this method is that extremely low parasite positive samples at the detection limit of expert microscopy are less likely to be included in the study sample. However, some RDTs did fail to register the samples with the lowest parasitaemia in the study as positive. In addition, three out of sixteen samples scored as negative by reference microscopy, which were also negative by all six RDTs, had positive PCR results.

Secondly, all RDT types and storage variants were performed in parallel for each sample and a high-resolution photograph of the panel was taken immediately after the results were read. This mechanism proved to be invaluable for quality assurance of the test results. RDTs may generate false positives if left for longer than the recommended reaction time, either due to decreased specificity of the reaction over time [54] or to the used test being adversely affected by humidity [55], so they cannot be stored for future independent examination. Photographic quality control provides a permanent record from which the types of invalid test and false positive or false negative results can be further categorized.

Finally, the use of the global performance index as a composite measure of test function in this study has greatly facilitated comparisons between the selected RDTs. Sensitivity and specificity analysis are typically used to evaluate diagnostic tests against a gold standard. However, these measurements can be misleading, as invalid test results are not included in these calculations and have to be reported separately. The number of invalid tests is an important consideration for at-scale use of these tests, because they could result in significant resource wastage and loss of faith in the device by end users. Thus, although the CareStart Pf/Pan test performed better than the others by any of the measurement criteria used, differences in sensitivity between this test and the Bioline test are quite moderate. If sensitivity and specificity alone were considered, it would be easy to draw the false conclusion that the two types of RDT have comparable performance; while in fact the Bioline test, if the invalid rate is taken into account, scores considerably lower.

Lastly, the global performance index may be a more appropriate measure to use when, as in this case, the proportion of true negative and true positive samples are not equivalent. Although intra-reader and intra-lot variation were not investigated in this study, the global performance index could also be expanded for use in this context, by comparing or averaging the score for multiple readers or test lots.

### Study limitations

This study was designed as a precursor to larger randomized trials of malaria rapid diagnostic tests in Afghanistan that are currently being conducted by the authors. Due to the short nature of this pre-study, there are a number of limitations which should be borne in mind when considering the implications of the results.

First, the samples that were selected for the study were predominantly *P. vivax *positive. This was done to ensure speedy recruitment that would aid in the determination of test sensitivity, as the prevalence of malaria in the study setting is low and *P. vivax *is the predominant species. Since the sample only included 20 malaria negative patients, the results cannot be used to infer the specificity of the RDTs. This phenomenon requires further investigation with a larger number of negative samples and some *P. falciparum *positive samples.

Secondly, patient recruitment was based on slide positivity according to the seven clinic microscopists. Three of the microscopists were expert-level and had been involved in other studies, while the remaining four had not been involved in a research programme before. The resultant sample is, therefore, restricted by their collective limit of detection and may not have included very low parasitaemic samples that were below this limit. This may be the reason why very few *P. vivax *positive samples in the study had parasitaemia less than 200/μL.

Thirdly, inter-lot variation in test performance was not investigated in this study, since in each case a single lot was evaluated. The points of concern raised here (poor sensitivity of the CareStart PfPv test to low parasitaemia and the high invalid rate of the Bioline PfPv device) may not be a consistent issue across lots. Further investigations with different lots of the same test models are needed to confirm if these issues are persistent.

Finally, although the evaporative cooler box performed well in this study, it had little effect on the performance of tests that were stored within it. This may be due to the fact that the study was conducted in a single location, with a small number of rapid tests (306). As such, this limits inferences that can be made on the impact of evaporative cooler boxes, should they be employed at-scale, where a greater protective effect may yet be observed.

## Conclusions

The CareStart Pf/Pan RDT outperformed the other two types of RDT evaluated on all parameters measured and gave reliable results, irrespective of storage conditions or the parasitaemia level in test samples. The results show that this RDT is appropriate for vivax-predominant regions, although the need for further evaluation against falciparum cases in this setting is recognized.

Conversely, the two RDTs that were affected by parasitaemia and/or storage conditions yielded important information; firstly, that poor sensitivity to a low parasitaemia may not only be due to heat damage and secondly that the mechanical operation of rapid tests may be adversely affected by heat.

The evaporative cooler box is an appropriate storage device for poorly resourced heath centres and its effectiveness in maintaining temperatures below the recommended maximum for RDTs has been demonstrated in this study.

Finally, this investigation has highlighted the utility of evaluating RDTs in parallel with field-collected venous blood samples from a mainly malaria-positive study population, using a composite measure of performance as the primary method of comparison. Taken together, these measures constitute a simple model for semi-controlled RDT field trials which bridge the gap between assessments of efficacy and effectiveness and may be usefully replicated in other countries.

## Competing interests

The authors declare that they have no competing interests.

## Authors' contributions

AFWM conceptualized the study, designed the protocol, analysed the data and drafted the manuscript. TJL designed the evaporative cooler box, contributed to the study design, analysis of the results and drafting of the manuscript. MIM helped design the study protocol, trained the field staff, supervised the field work and contributed to analysis of the results. RZ performed the Mangold malaria PCRs and interpreted the results. NM contributed to the study design, facilitated and monitored the field work, and critically reviewed the manuscript. MAH supervised the field work and contributed to analysis of the results. NS contributed to the study design, facilitated the execution of the study at the Malaria Reference Centre and critically reviewed the manuscript. CJMW contributed to the study concept and design and critically reviewed the manuscript. MR contributed to the study concept and design, analysis and interpretation of the results and drafting of the manuscript. All authors have read and approved the final manuscript.
